# Molecular Aspects of Medicinal Fungi

**DOI:** 10.3390/jof8020138

**Published:** 2022-01-29

**Authors:** Jen-Tsung Chen

**Affiliations:** Department of Life Sciences, National University of Kaohsiung, Kaohsiung 811, Taiwan; jentsung@nuk.edu.tw

For thousands of years, fungi or mushrooms have been used as biological control agents, cosmeceuticals, dietary foods and supplements, pharmaceuticals, and traditional medicines around the world. It is well-recognized that natural compounds and enzymes from fungi possess a range of bioactivities and medicinal functions, including antibacterial, antioxidant, antitumor, antiviral, and dermal protective activities, immunomodulation, etc. Nowadays, medicinal fungi are a critical source for pharmaceuticals; however, our knowledge about the molecular mechanisms underlying their biological or medical actions is still limited. The aim of this Special Issue, entitled “Molecular Aspects of Medicinal Fungi”, is to explore insights into the efficacy of natural compounds from fungi, publishing three original research papers and two literature reviews.

Fungi from the marine environment are an abundant source of bioactive compounds that exert anticancer, antibiotic, and antiviral activities, among others. Noman et al. [1] contribute a systematic literature review summarizing the sources and underlying mechanisms for antitumor activities from natural marine products. It was concluded that in marine fungi, actinomycin, doxorubicin, and flavonoids are primary sources of drug candidates for treating breast, colon, colorectal, ovary, pancreatic, and uterine cervix cancers.

Fungi-derived polysaccharides have been studied for their regulatory activities in the immune system. Vetvicka et al. published a review article describing the effects of β-glucans, with emphasis on the mechanisms of immunomodulatory action and tumor progression [2]. The authors concluded that among fungi-derived β-glucans, several compounds such as lentinan, were proven to have considerable anticarcinogenic and immunostimulatory effects. They proposed that further long-term studies involving clinical trials are needed to clarify the health benefits from β-glucan-containing fungal foods.

Red yeast rice, red koji rice, or Anka is made by the fermentation of *Monascus* species for food products that have been popular in Asia for over one thousand years. Wu et al. isolated and identified xanthonoid and naphthalenone components from *M. purpureus* BCRC 38110 [3]. Among these, monascuspirolide B and ergosterol peroxide exert dermal protection activities and are suggested to be potential candidates for developing cosmeceutical products in the future.

Honey mushroom, *Armillaria mellea* (Tricholomataceae), has a symbiotic relationship with the significant herbal medicinal plant, *Gastrodia elata*, and could be used as its alternative for treating neurological diseases. Li et al. investigated the effects of water and ethanolic extracts from *A. mellea* mycelium on sleep behavior, using rats as a model system [4]. Finally, the authors identified γ-aminobutyric acid (GABA) and seven sesquiterpenoids from the extracts and demonstrated that the extracts exerted sleep promotion activity.

Members of the *Pleurotus* genus are edible mushrooms, and are used in Asian folk medicines for treating cancers, immunological disorders, wounds, etc. Maury et al. investigated the effect of *P. ostreatus* mycelium extract, finding that it has immunomodulatory potential for macrophage-mediated innate immune responses [5]. The authors suggested that the extract could be developed further for a complementary therapy on immune dysfunction or for maintaining homeostasis of the immune system.

Altogether, the findings of this Special Issue enrich our understating of bioactivities from the components of edible and medicinal fungi and fermented products, and it is just a snapshot of the rapid development in this interesting and critical research field. In the future, the insights and applications of natural compounds from fungi will be revealed more efficiently with the assistance of advanced tools, high-throughput technologies, and emerging biotechnologies such as drug-delivery systems, genome editing, multi-omics, nanotechnology, and systems pharmacology.

## Data Availability

Not applicable.
